# Altered microRNA expression profile with miR-146a upregulation in CD4^+ ^T cells from patients with rheumatoid arthritis

**DOI:** 10.1186/ar3006

**Published:** 2010-05-11

**Authors:** Jingyi Li, Ying Wan, Qiuye Guo, Liyun Zou, Jinyu Zhang, Yongfei Fang, Jingbo Zhang, Jinjun Zhang, Xiaolan Fu, Hongli Liu, Liwei Lu, Yuzhang Wu

**Affiliations:** 1 Institute of Immunology, PLA, Third Military Medical University, 30# Gaotanyan Street, District Shipingba, Chongqing 400038, PR China; 2 Department of Rheumatology, Southwest Hospital, Third Military Medical University, Chongqing, 30# Gaotanyan Street, District Shipingba, Chongqing 400038, PR China; 3 Department of Pathology and Center of Infection and Immunology, The University of Hong Kong, Pokfulam Road, Hong Kong, PR China

## Abstract

**Introduction:**

Increasing evidence indicates that microRNAs (miRNAs) play a critical role in the pathogenesis of inflammatory diseases. The aim of the study was to investigate the expression pattern and function of miRNAs in CD4^+ ^T cells from patients with rheumatoid arthritis (RA).

**Methods:**

The expression profile of miRNAs in CD4^+ ^T cells from synovial fluid (SF) and peripheral blood of 33 RA patients was determined by microarray assay and validated by qRT-PCR analysis. The correlation between altered expression of miRNAs and cytokine levels was determined by linear regression analysis. The role of miR-146a overexpression in regulating T cell apoptosis was evaluated by flow cytometry. A genome-wide gene expression analysis was further performed to identify miR-146a-regulated genes in T cells.

**Results:**

miRNA expression profile analysis revealed that miR-146a expression was significantly upregulated while miR-363 and miR-498 were downregulated in CD4^+ ^T cells of RA patients. The level of miR-146a expression was positively correlated with levels of tumor necrosis factor-alpha (TNF-α), and in vitro studies showed TNF-α upregulated miR-146a expression in T cells. Moreover, miR-146a overexpression was found to suppress Jurkat T cell apoptosis. Finally, transcriptome analysis of miR-146a overexpression in T cells identified Fas associated factor 1 (FAF1) as a miR-146a-regulated gene, which was critically involved in modulating T cell apoptosis.

**Conclusions:**

We have detected increased miR-146a in CD4^+ ^T cells of RA patients and its close correlation with TNF-α levels. Our findings that miR-146a overexpression suppresses T cell apoptosis indicate a role of miR-146a in RA pathogenesis and provide potential novel therapeutic targets.

## Introduction

Rheumatoid arthritis (RA) is a common chronic inflammatory disease characterized by radiographic joint destruction with severe functional deterioration and high mortality. A hallmark pathological feature of RA is the infiltration and accumulation of T cells in the synovium of joint [1]. As the shared epitope in human leukocyte antigen-DR genes is found in about 80% of RA patients, dysregulated CD4^+ ^T cell activation and function have been investigated based on the available evidence of genetic predisposition [2,3].

T cells isolated from joint tissue and synovial fluid (SF) show an activated and memory phenotype and appear to respond poorly to stimulation with mitogen or antigens *in vitro *[4,5]. These T cells are unusually resistant to apoptosis in SF that contains a significant amount of pro-apoptotic factors such as bioactive FasL, TRAIL and TNF-α [6,7]. In addition, studies on a murine model of proteoglycan-induced arthritis also showed that CD4^+ ^T cells failed to undergo apoptosis [8]. All these findings from patients and animal models suggest that the inhibition of T cell death may result in the persistence and accumulation of T cells in synovium, as well as the accumulation of T cells in the periphery. The long-term survival of CD4^+ ^T cells has been shown to affect the behavior of synovial fibroblasts through the cell-to-cell contact and the secretion of proinflammatory factors such as Th1 and Th17 cytokines [9,10], ultimately contributing to the maintenance and exacerbation of inflammation in RA [11]. Although elevated levels of anti-apoptotic proteins such as the Bcl-2 family have been found in these T cells [12], the possible mechanism underlying the impaired apoptosis of T cells in RA remains largely unclear.

MicroRNAs (miRNAs) are about 22 nucleotide (nt) non-coding RNA that regulate mRNA expression at the posttranscriptional level for degradation or translational repression, which have been found to control cell division, differentiation and death [13]. To date, thousands of miRNAs have been identified in mammalian genomes, and up to 30% of human genes are regulated by them [14]. Recently, miRNAs have been recognized as a novel player in normal immune function and inflammation [15]. In particular, T cell-mediated immune responses are associated with changes in the expression of specific miRNAs. CD4^+ ^T cells have also been found to express different miRNAs subsets that are linked to cell differentiation, maturation, activation and function [16-19]. Notably, a growing number of reports have revealed that deregulation of miRNA expression contributes to human autoimmune diseases including psoriasis and systemic lupus erythematosus [20,21], in which expression of a set of altered miRNAs are identified. In RA patients, increased expression of miR-146a, miR-155, miR-132 and miR-16 have been found in peripheral blood mononuclear cells (PBMCs) [22]. Recently, analysis of miRNA expression profile has revealed that miR-223 is overexpressed in peripheral T cells of RA patients [23].

Moreover, there is evidence that proliferation of fibroblast-like synoviocytes is regulated by miR-124a [24]. The abnormal expression of miR-146 and miR-155 is also found in the synovium of RA patients, while the expression of these miRNAs is markedly upregulated in SF after stimulation with TNF-α and IL-1β [25,26], which indicate that the inflammatory milieu may alter the miRNAs expression in joint tissue.

Although a set of altered expression miRNAs have been identified in synovial tissue or PBMC of patients with RA, the functions of these dysregulated miRNAs remain largely unclear. In particular, both the expression profile and the roles of miRNAs in CD4^+ ^T cells of RA patients have not been characterized. In this study, we revealed that miR-146a expression was upregulated in CD4^+ ^T cells from RA patients, exhibiting a close correlation with increased levels of TNF-α. Furthermore, we demonstrated that the increased expression of miR-146a suppressed T cell apoptosis by regulating Fas-associated factor 1 (FAF1). These findings suggest that miR-146a in CD4^+ ^T cells may play an important role in RA pathogenesis.

## Materials and methods

### Patients and control samples

All patients in the study who first visited at Rheumatology Department of Southwest Hospital fulfilled the American College of Rheumatology criteria for the classification of RA. All patients in this study had been diagnosed without other complications. The patients had never been treated with oral or intra-articular corticosteroids, disease-modifying antirheumatic drugs (DMARDs) or other immunosuppressive drugs. However, nonsteroidal anti-inflammatory drugs and other symptomatic treatments were given to some patients. Samples from healthy donors were collected from 12 volunteers, half of which were men and half women. Serum and peripheral blood samples were collected from RA patients and healthy donors. SF from RA patients was collected by routine knee joint paracentesis. At least 10 ml of SF and 20 ml of peripheral blood anticoagulated by heparin were collected.

All study protocols and consent forms were approved by the Institutional Medical Ethics Committee of Third Military Medical University. Written permission was obtained from all subjects who participated in the study.

### Microarray analysis

CD4^+ ^T cells were purified from the SF of two RA patients and peripheral blood of one healthy donor using microbeads (MACS, Miltenyi Biotec, Bergisch Gladbach, Germany). Total RNA of these CD4^+ ^T cells was prepared using the mirVana miRNA Isolation Kit (Ambion, Austin, Texas, USA) for miRNA microarray analysis and real-time RT-PCR assay. Hybridization was carried out using a miRCURYTM Array microarray kit (Exqion, Vedbaek, Denmark), which contained 461 mature miRNA probes. The Affymetrix GeneChip (Human Genome U133 Plus 2.0 Array, Affymetrix, Santa Clara, CA) was used to analyze over 47,000 transcripts of Jurkat T cells before and after transfection with FUGW-FF3, FUGW-miR-146a or FUGW-miR-146a sponge. The expression data and gene annotations were stored in NCBI's Gene Expression Omnibus (GEO) database, accession no. [GEO:GSE21118] and [GEO:GSE21132] [27].

### Quantitative RT-PCR analysis

Quantitative RT-PCR assays were performed using a TaqMan^® ^MicroRNA Assays kit (Applied Biosystems, Carlsbad, CA, USA) for the mature miRNA according to manufacturer's instructions. RUN6B small nuclear RNA was quantified as a control to normalize differences in total RNA levels. Specific primers to FAF1 for real-time PCR were designed using Roche's Applied Science Universal Probe Library Assay Design Center (Roche, Indianapolis, Indiana, USA) [28]; β-actin was quantified as the control. The primer sequences for FAF1 were as follows: 5'-CAGCGGGAGTACAACCTGA-3' (forward) and 5'-AAGGTCATACACATTTCTCTTTACCTC-3' (reverse).

The quantitative RT-PCR was performed by using Opticon-2 Detection System (Applied Biosystems, Carlsbad, CA, USA). All reactions were run in duplicate and repeated three times. A threshold cycle was determined in the exponential phases of amplification, and the comparative threshold cycle method was calculated with 2^ΔΔT ^and used to calculate the relative gene expression. The value of each control sample was set at one and was used to calculate the fold change in target genes.

### Cloning of miR-146a gene and construction of vectors

A 452 bp sequence containing the miR-146a hairpin (nucleotides 4721752-4722203 of chromosome5-EMBL reference) was amplified from human genomic DNA by PCR. The primer sequences for primary miR-146a were as follows: 5'-AATGCGGCCGCTCAAGAGATCCACCCACATC-3' (forward) and 5'-CCGACGCGTGCTACTTGGAACCCTGCTTA-3' (reverse). The miR-146a-coding genomic DNA fragment was cloned downstream from the GFP gene of the lentiviral vector FUGW by digesting with BsrGI and EcoRI. Similarly, FAF1 cDNA (Open Biosystems, Huntsville, Alabama, USA) was cloned into FUGW. The plasmids were verified by sequencing.

### Lentivirus production and transfection

Lentivirus particles derived from HIV were produced by transient co-transfecting lentiviral expression constructs into 293FT cells with pFIV-PACK Lentiviral Packaging Kit (System Biosciences, Mountain View, CA, USA) following Ca_3_(PO4)_2 _transfection protocol. The production of HIV replication incompetent lentiviral particles was performed by simultaneously delivering lentiviral transfer vectors and packaging plasmids (pSPAX2 and pMD2.G) into 293FT cells. Pseudo-viral particles generated by 293 FT cells within 48 hours were centrifuged at 100,000 g for two hours and frozen at -70°C for later experiments. Jurkat T cells were seeded into 24-well plates and lentivirus was used to transduce at a multiplicity of infection (MOI) of 10 for 48 hours. Cells were analyzed by FACs and quantitative RT-PCR.

### Cytokines assay

The BD™ Cytometric Bead Array Human Th1/Th2 Cytokine kit (BD Biosciences, San Jose, CA, USA) was used to quantitatively measure the levels of IL-2, IL-4, IL-6, IL-10, TNF-α and interferon (IFN)-γ in serum and SF according to manufacturer's instructions. CD4^+ ^T cells from healthy donors were transfected with FUGW-FF3 and FUGW-mi-R146a, then stimulated with Phorbol myristate acetate (PMA) (50 ng/ml) and ionomycin (1 μg/ml). Levels of IL-2, IL-17A, TNF-α and IFN-γ expression were detected by intracellular staining. Cytoplasmic expression of these cytokines was determined in the green fluorescent protein (GFP^+^) population by FACs. All samples were analyzed on a FACS Aria flow cytometer and all data were analyzed by FlowJo software (BD Biosciences, San Jose, CA, USA).

### Cell stimulation

CD4^+ ^T cells were sorted using microbeads (MACS, Miltenyi Biotec, Bergisch Gladbach, Germany). The quantity of CD4^+ ^T cells should be at least half million and the purity should be at least 90%. Jurkat cell line was purchased from ATCC (Manassas, VA, USA). SF from 10 patients were mixed and centrifuged. The supernatant of SF mixture was collected. Then Jurkat cells and normal CD4^+ ^T cells were stimulated by SF diluted to 1:2 or TNF-α for 48 hours.

### Evaluation of cell proliferation and apoptosis

Jurkat T cells transfected with FUGW-FF3 or FUGW-miR-146a were stimulated with PHA (30 μg/ml) for the indicated time. Cell proliferation was assayed with a cell counting kit-8 [29] (CCK-8, Dojindo, Kumamoto, Japan). Briefly, 10 μl of CCK-8 solution was added to each well of the plate, and the plate was incubated at 37°C for one hour. The absorbance was measured at 450 nm as an indicator of cell viability. All experiments were independently repeated three times. FUGW-FF3, FUGW-miR-146a and FUGW-miR-146a-FAF1 Jurkat T cells were grown in RPMI with 3% FBS and stimulated with anti-Fas antibody (100 ng/ml) for six hours. Cells were stained with Annexin V and propidium iodide (PI) for flow cytometric analysis.

### Statistical analysis

All data were analyzed by SPSS10.0 software (SPSS, Chicago, IL, USA). Microarray data were analyzed by Cluster Analysis. All data were represented by mean values ± standard deviation. *P *values less than 0.05 were considered statistically significant. Independent simple T test was used to compare with different groups. The relation between miRNA and clinical demographics was analyzed by Pearson Correlation.

## Results

### Increased miR-146a expression in CD4^+ ^T cell from SF and peripheral blood of RA patients

**Figure 1:**
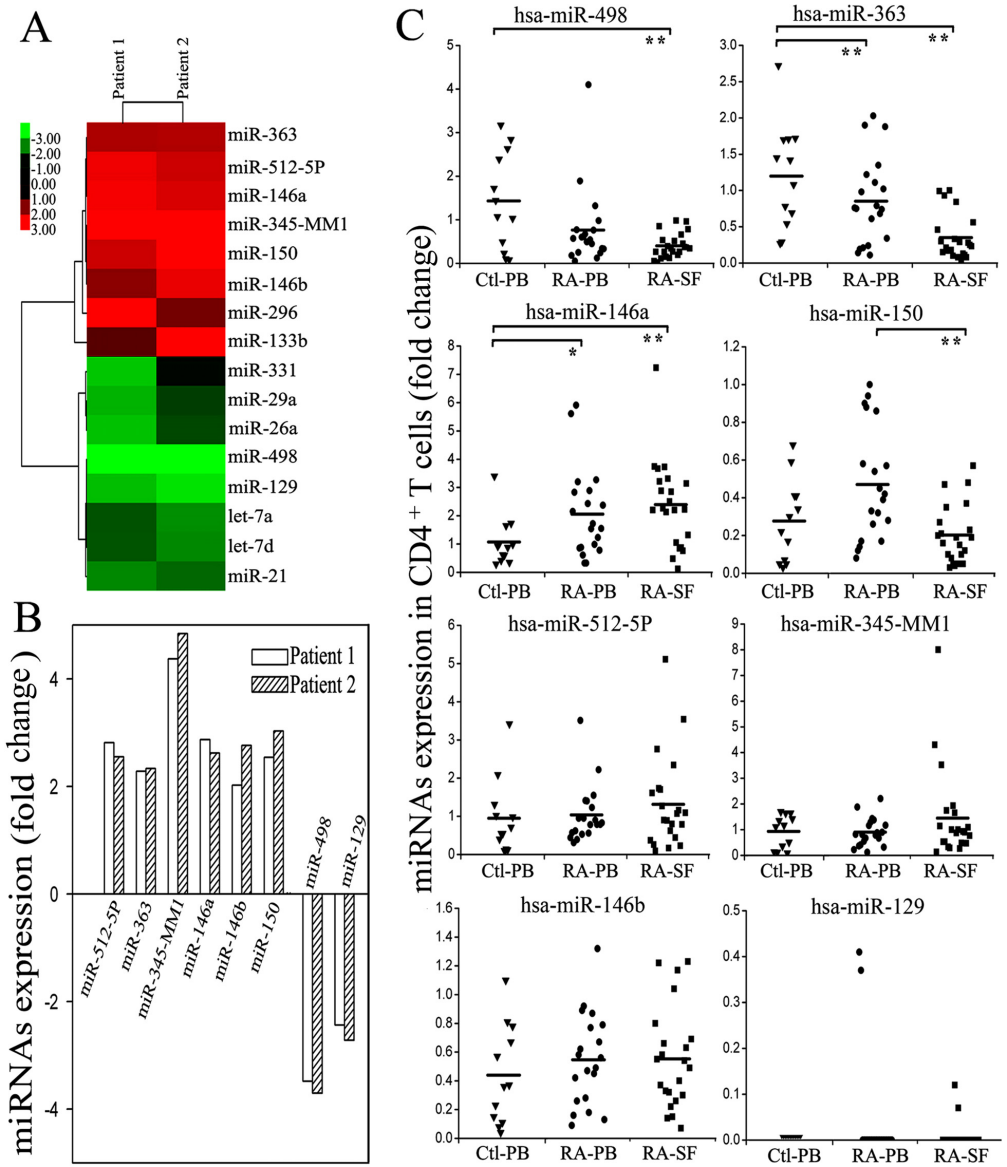
**The altered expression profile of miRNAs in CD4^+ ^T cells from SF of RA patients**. **(a) **Compared with normal CD4^+ ^T cells, altered expression of 16 microRNAs (miRNAs) with fold change (>2) was found in CD4^+ ^T cells of rheumatoid arthritis (RA) by miRNA microarray analysis. (red: upregulation; green: downregulation) **(b) **Expression of eight miRNAs was altered in both RA patients by miRNA microarray analysis. Six of them were upregulated and two of them were downregulated. **(c) **Compared with healthy controls, levels of miRNAs - miR-498, miR-363, miR-146a, miR-150, miR-512-5P, miR-345-MM1, miR-146b and miR-129 - expression in peripheral blood (PB; n = 20) and synovial fluid (SF; n = 22) of RA patients and PB (n = 12) of healthy controls were determined by Taqman quantitative RT-PCR analysis. Horizontal bars represent the mean values. Ctl-PB: CD4^+ ^T cells from PB of healthy controls; RA-PB: CD4^+ ^T cells from PB of RA patients; RA-SF: CD4^+ ^T cells from SF of RA patients. **P < 0.01, *P < 0.05.

The expression profiles of miRNAs in CD4^+ ^T cells of SF from two RA patients were determined by miRNA microarray analysis. The clinical features and serological parameters of patients with RA were shown in Table 1. Compared with normal peripheral blood CD4^+ ^T cells, pairwise significance analysis of microarray data indicated that the expression levels of eight miRNAs (miR-363, miR-512-5P, miR-345-MM, miR-146a, miR-146b, miR-296, miR-133b and miR-150) were increased more than two-fold by a change in CD4^+ ^T cells of SF from RA patients whereas eight miRNAs (miR-331, miR-29a, miR-26a, miR-498, miR-129, let-7a, let-7d and miR-21) were significantly downregulated (Figure 1a). According to the results of microarray analysis, we focused on verifying the expression of those miRNAs whose expression levels were altered in both RA patients (Figure 1b).

**Table 1:** Demographics clinical features of RA patients

Parameter	Patient 1	Patient 2
Age (years)	19	48
Sex	Female	Female
Duration (months)	18	14
RF titer (IU)	74	82.3
ESR (mm/hours)	69	120
CCP titer (IU)	97	85
DAS28 (ESR) score	7.51	6.85

CCP, cyclic citrullinated peptide antibody; DAS28, Disease Activity Score using 28 joint counts; ESR, erythrocyte sedimentation rate; RA, rheumatoid arthritis; RF, rheumatoid factor.

To confirm the microarray data, eight miRNAs with altered expression levels in both patients (miR-512-5P, miR-363, miR-345-MM1, miR-146a, miR-146b, miR-150, miR-498 and miR-129) were analyzed in CD4^+ ^T cells from 33 patients by quantitative RT-PCR analysis. Both clinical features and serological parameters of these patients were shown in Table 2. Compared with CD4^+ ^T cells from peripheral blood of healthy donors, miR-146a expression was significantly increased in CD4^+ ^T cells from both peripheral blood and SF of RA patients, while miR-498 was only down-regulated in SF T cells from patients (Figure 1c).

**Table 2:** Demographics and clinical features of RA patients

Parameter	Value
Age, mean ± SD years	48 ± 16.5
Sex, male/female	8/33
Duration of symptoms, mean ± SD months	58.28 ± 51.84
RF positive, %	84.85%
RF titer, median units(range)	117.4(20-1060)
ESR, mean ± SD mm/hours	67.94 ± 21.19
CCP titer, median units(range)	74.5(15-800)
Stiffness, median minutes(range)	76.67 ± 91.31
DAS28 (ESR) score, mean ± SD	6.35 ± 1.45

CCP, cyclic citrullinated peptide antibody; DAS28, Disease Activity Score using 28 joint counts; ESR, erythrocyte sedimentation rate; RA, rheumatoid arthritis; RF, rheumatoid factor; SD, standard deviation.

### Positive correlation of increased miR-146a expression in T cell with elevated TNF-α levels

**Figure 2:**
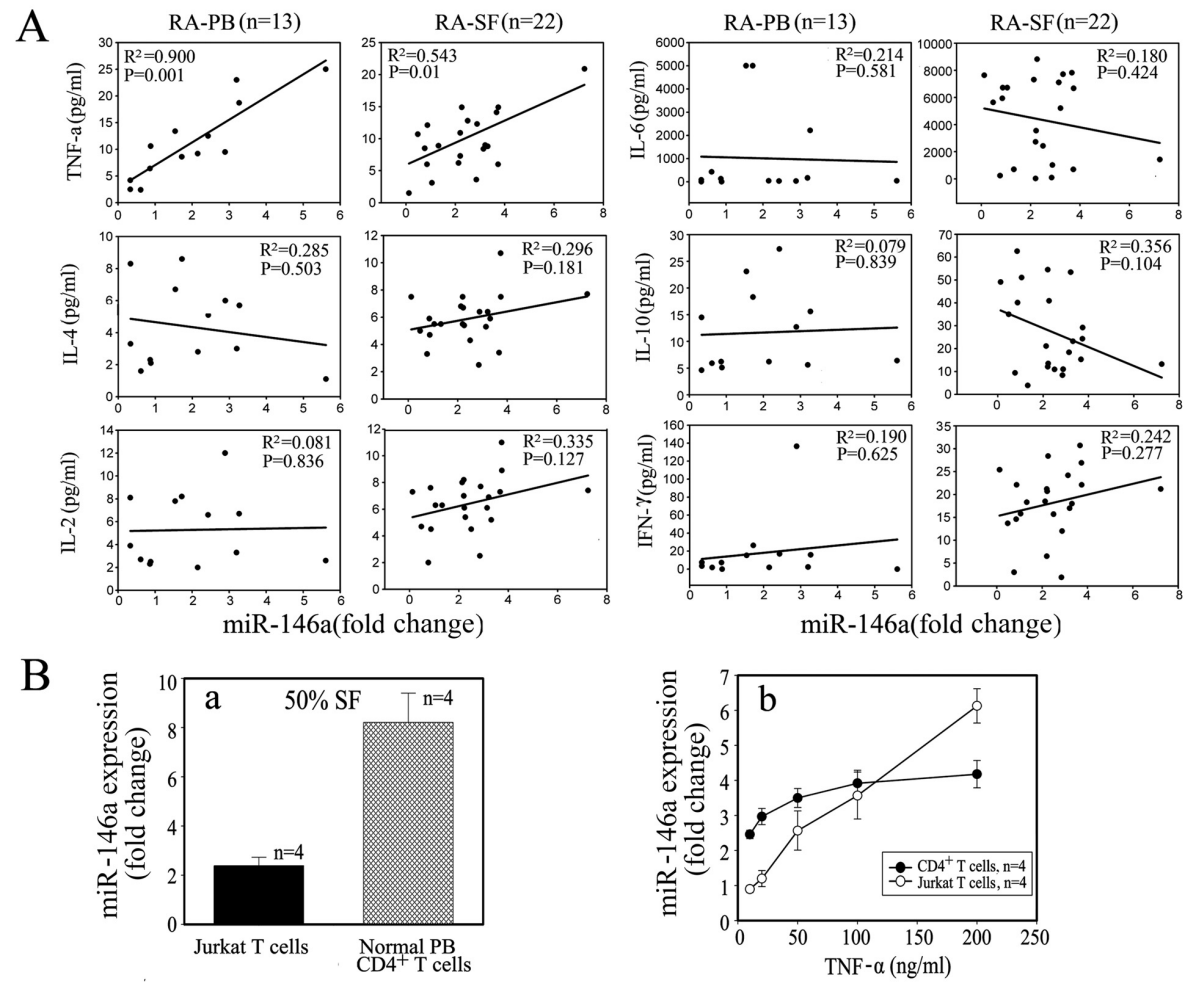
**Positive correlation of miR-146a expression with TNF-α levels in RA patients**. **(a) **A positive correlation was found between miR-146a expression and TNF-α levels. Of synovial fluid (SF), 50% increased the expression of miR-146a in Jurkat T cells by 2.39 ± 0.34 fold and in human CD4^+ ^T cells by 8.21 ± 1.19 fold (n = 4), respectively. **(b) **Induction of miR-146a expression stimulated with TNF-α or SF of rheumatoid arthritis (RA) in both Jurkat cells and CD4^+ ^T cells. Unstimulated Jurkat T cells or CD4^+ ^T cells were used as controls, respectively. The value of each control sample was set at one and further used to calculate the fold change. TNF-α upregulated miR-146a expression in Jurkat T cells and human CD4^+ ^T cells in a dose-dependent fashion.

To determine the possible correlation of these altered miRNAs with secreted cytokines in RA patients, we measured the levels of IL-2, IL-4, IL-6, IL-10, IFN-γ and TNF-α in both SF and serum of RA patients and healthy donors [Supplemental figure S1 in Additional file 1], among which the levels of TNF-α were found to be significantly higher in SF and serum of RA patients than healthy donors. The linear regression analysis showed that only miR-146a expression was positively correlated with the levels of TNF-α in both peripheral blood and SF of RA patients, but not correlated with the levels of other cytokines (Figure 2a) and disease activity indexes such as rheumatoid factor, anti-cyclic citrullinated peptide antibody, erythrocyte sedimentation rate, C-reactive protein and disease activity score of 28 joints (DAS28) scores (data not shown). Next, Jurkat T cells and normal CD4^+ ^T cells were stimulated with SF and TNF-α *in vitro*, and expression of miR-146a was found to be upregulated in a dose-dependent fashion (Figure 2b). Moreover, the expression of miR-498, miR-363 and miR-150 did not show any correlation with levels of cytokine production [Supplemental figure S2 in Additional file 2]. Thus, these data indicate that miR-146a may play a significant role in inflammation of RA.

### Overexpression of miR-146a suppresses T cell apoptosis but does not alter cytokine production

**Figure 3:**
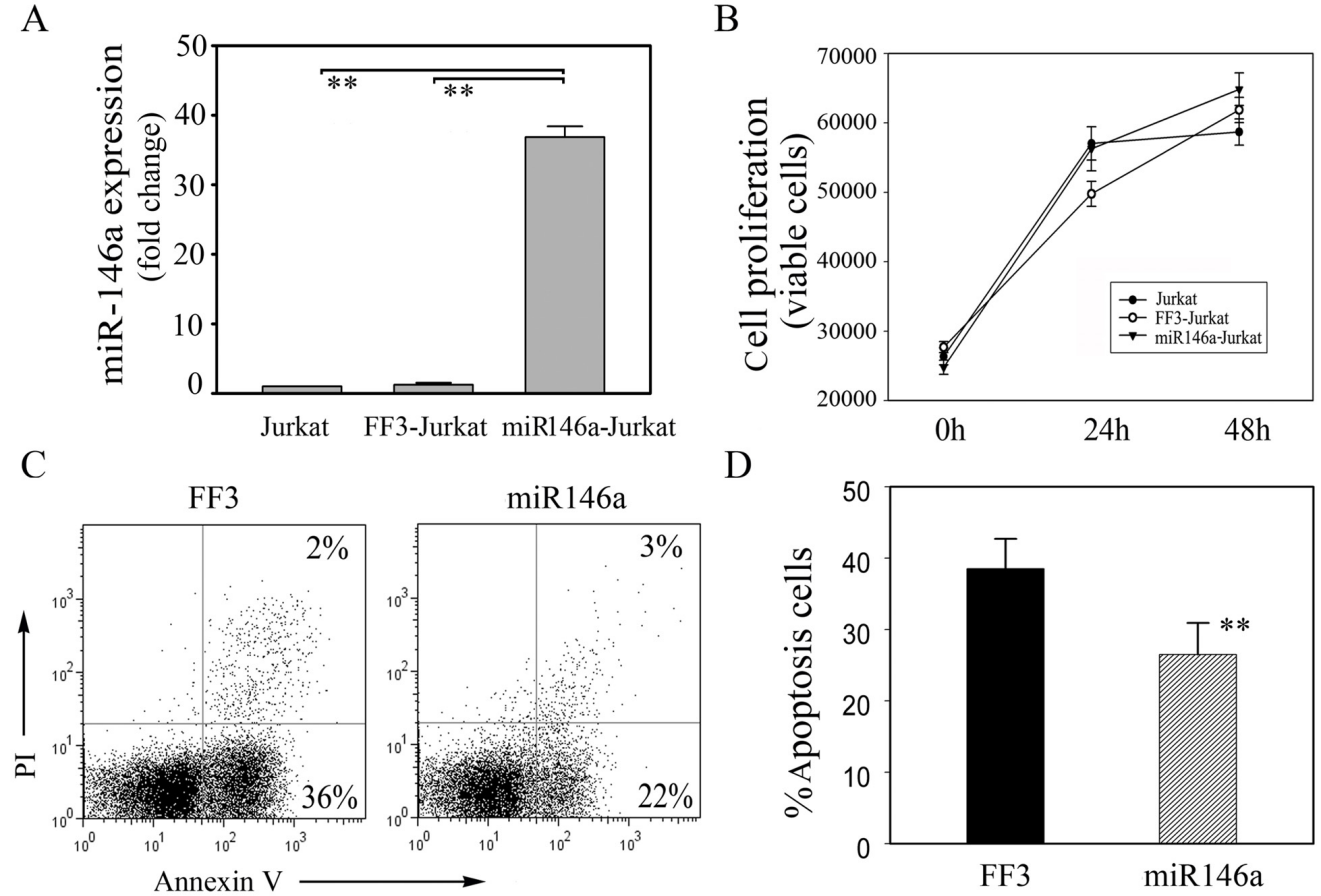
**Overexpression of miR-146a suppresses T cell apoptosis**. **(a) **The expression of miR-146a in Jurkat T cells measured by quantitiative RT-PCR increased more than 30 fold after transfection with FUGW-miR-146a, but showed no change after transfection with the control vector FUGW-FF3. **(b) **After phytohemagglutinin (PHA) stimulation, Jurkat T cells with miR-146a overexpression showed a similar proliferative response as their controls. **(c) **Upon anti-Fas antibody treatment for six hours, miR-146a-overexpressing Jurkat T cells displayed significantly reduced apoptosis as detected by flow cytometry. **(d) **After six hours of anti-Fas antibody treatment, apoptotic Jurkat T cells were evaluated. The data are derived from three independent experiments (mean ± standard deviation). **P < 0.01.

To investigate the potential role of miR-146a in T cell functions, we amplified the coding region of miR-146a and inserted it into the lentivirus vector FUGW, in which the ubiquitin promoter drove the expression of GFP and miR-146a. Moreover, the vector FUGW-FF3 containing GFP and miR30-shRNA served as a negative control. Upon transfection with high efficiency, Jurkat T cells were found to express miR-146a 30 times more than controls (Figure 3a). PHA stimulation, Jurkat T cells with miR-146a overexpression showed a similar proliferative response as their controls (Figure 3b). However, miR-146a-overexpressing Jurkat T cells displayed significantly reduced apoptosis in culture (Figure 3c). To further verify the effect of miR-146a overexpression on cytokine production in primary T cells, we transfected freshly prepared CD4^+ ^T cells from peripheral blood of healthy donors and analyzed the profile of cytokine production after PMA stimulation. Flow cytometric analysis did not detect any significant alteration in the expression levels of IFN-γ, TNF-α, IL-2 and IL-17A in normal CD4^+ ^T cells transfected with miR-146a [Supplemental figure S3 in Additional file 3].

### miR-146a overexpression suppresses T cell apoptosis possibly by regulating FAF1 expression

**Figure 4:**
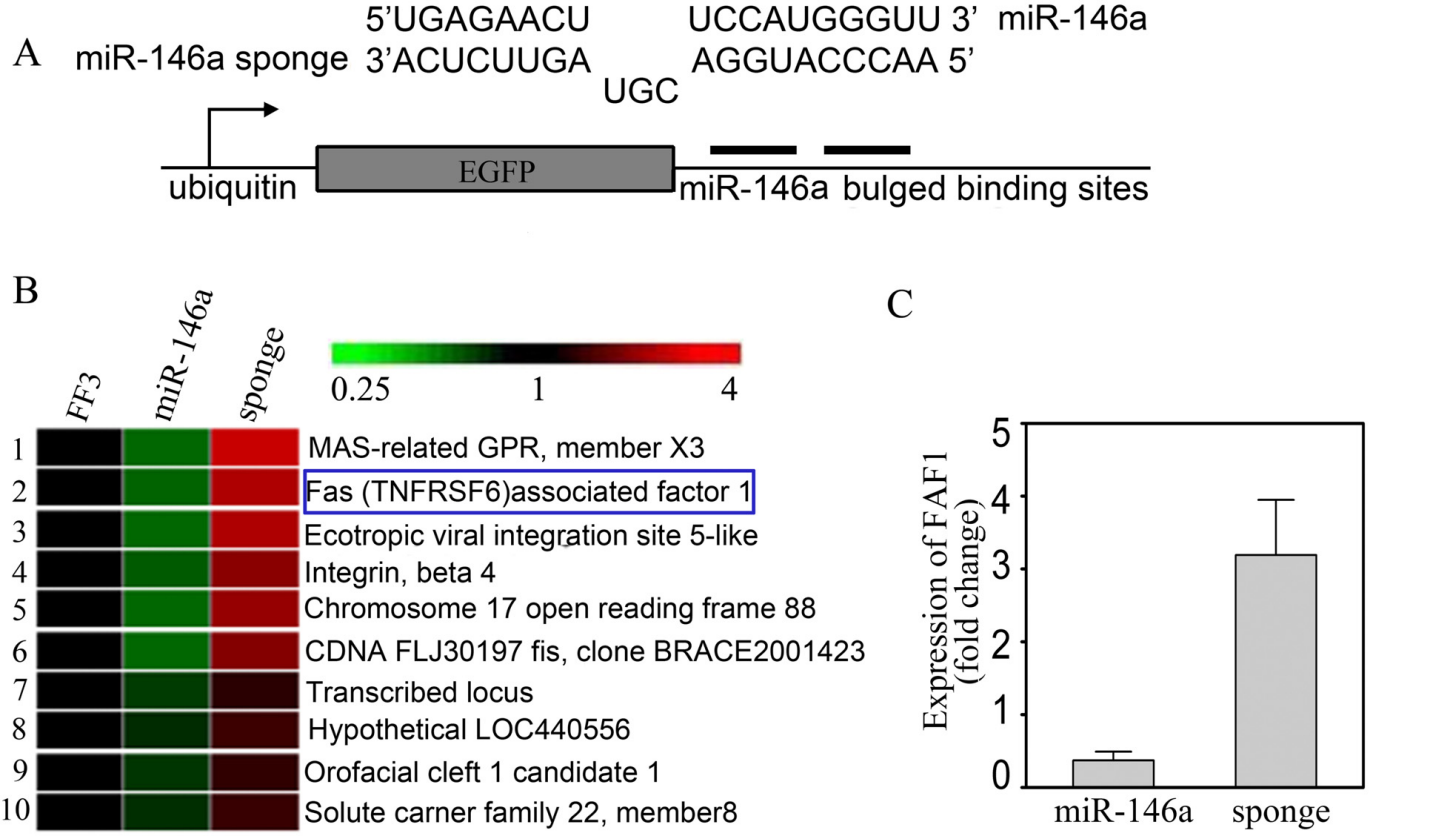
**Gene expression profiles in Jurkat T cells with up-regulated and down-regulated miR-146a expression**. **(a) **Schematic representation of FUGW-miR-146a sponge, which was inserted into lentivirus vector FUGW. **(b) **Gene expression profiles of Jurkat T cells transfected with FUGW-miR-146a or FUGW-miR-146a sponge were analyzed by Affymetrix GeneChip. Fas-associated factor 1 (FAF1) was found to be negatively correlated to the level of miR-146a. **(c) **Expression levels of FAF1 were further verified by quantitative RT-PCR, consistent with the results from gene chip analysis. FUGW-FF3-Jurkat T cells were used as the control. The value of each control sample was set at one and further used to calculate the fold change.

**Figure 5:**
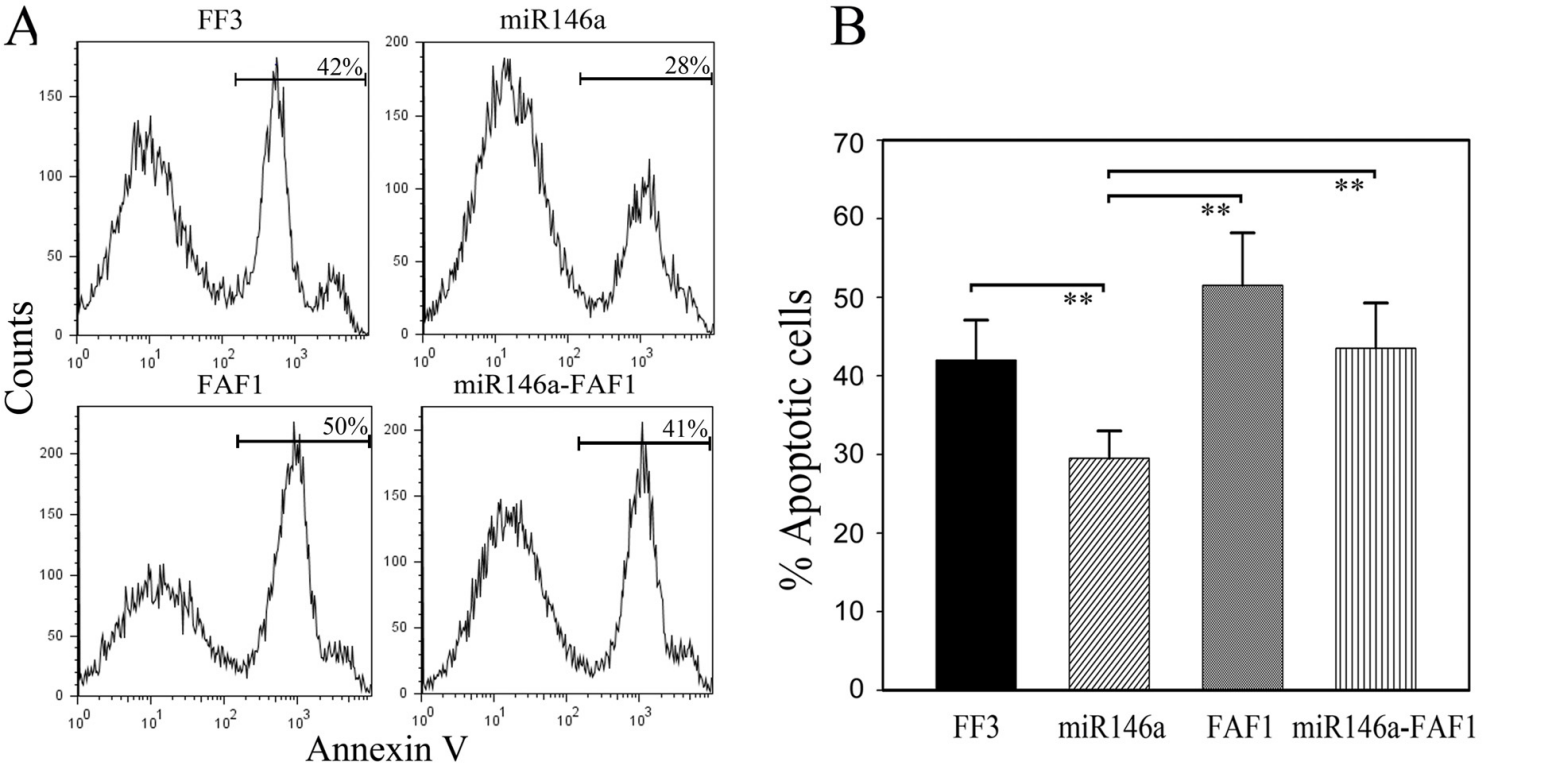
**FAF1 abolishes the suppressive effect of miR-146a on Jurkat T cell apoptosis**. **(a) **Fas-induced apoptosis was evaluated in Jurkat T cells transfected with either miR-146a alone or together with Fas-associated factor 1 (FAF1). miR-146a-overexpressing T cells showed significantly reduced apoptosis whereas co-overexpressing FAF1 abrogated the effect of miR-146a on T cell apoptosis. **(b) **After six hours of anti-Fas treatment, apoptotic Jurkat T cells were evaluated. The data are derived from three independent experiments (mean ± standard deviation). **P < 0.01.

To search for the potential target genes of miR-146a, we used the miRNA sponge technique employing miRNA specific decoy targets to suppress endogenous miRNA activity without affecting its transcription. We constructed the miR-146a sponge by inserting two miR-146a binding sites into the lentivirus vector FUGW with a reporter gene-eGFP driven by the ubiquitin promoter. The sponge had an imperfect binding site for miR-146a seed region, with a bulge at positions 9 to 12, which can prevent endonucleolytic cleavage (Figure 4a). We transfected the miRNA sponge into Jurkat T cells and compared the gene expression profiles when miR-146a expression was up- and down-regulated. Together, six genes were found to be regulated by altered levels of miR-146a expression (Figure 4b), among which the FAF1 expression was negatively correlated with the levels of miR-146a as further confirmed by quantitative RT-PCR analysis (Figure 4c). Next, FAF1 was cloned into lentivirus vector FUGW and plasmid FUGW-miR-146a and transfected into Jurkat T cells. We further evaluated Fas-induced apoptosis of Jurkat T cells when transfected with either miR-146a alone or together with FAF1. It was found that miR-146a-overexpressing T cells showed significantly reduced apoptosis while co-overexpressing FAF1 abrogated this effect, indicating that miR-146a suppresses apoptosis possibly via regulating the expression of FAF1 gene in T cells (Figure 5).

## Discussion

Increasing evidence indicates that aberrant expression of miRNAs is implicated in the pathogenesis of autoimmune diseases. Accumulating data have suggested that the proper regulation of miRNA expression is important in the maintenance of normal immune functions and prevention of autoimmunity. A number of miRNAs have been found to show a tissue-specific pattern during cancer development and viral infection. Previous studies have shown that increased miR-17-92 expression in lymphocytes leads to the development of lymphoproliferative disorder and autoimmune disease in mice [30]. Recently, upregulated miR-146a has been found in activated primary T cells and memory T cells [31]. Moreover, increased miR-146a expression has been detected primarily in PBMC and synoviocytes from RA patients [22,25]. Although deregulation of miRNA expression has been observed in human autoimmune diseases, it is still largely unclear how miRNAs affect autoimmune pathogenesis in patients. Although a set of altered expression miRNAs are recently identified in both PBMC and synovial tissue from RA patients, neither miRNAs expression profile nor their roles have been fully characterized in CD4^+ ^T cells of RA patients.

In this study, we have selected the RA patients who did not have any DMARDs therapy because it is known that drug treatment can affect expression of miRNAs [32]. We have found CD4^+ ^T cells from both SF and peripheral blood of RA patients exhibiting a specific miRNAs expression profile. Although miR-146a expression was not found to be associated with the disease activity index in the current study, our results have shown a significant positive correlation between miR-146a and TNF-α in both peripheral blood and SF by dependability statistical analysis. Furthermore, we have found that miR-146a expression is upregulated in CD4^+ ^T cells in response to TNF-α or SF stimulation *in vitro*. Thus the inflammatory milieu might alter miR-146a expression in infiltrated T cells. TNF-α is a critical mediator of the inflammatory pathway in the rheumatoid joints. As TNF-α inhibition therapy appears to dramatically reduce markers of inflammation and slow joint structural damage. Our findings of overexpression of miR-146a in T cells may have functional implications in eliciting joint inflammation of RA patients.

In addition to its increasingly recognized function in modulating innate immunity, miR-146a has been shown to be involved in Th1/Th2 polarization and regulatory T cell development [33-36], indicating a potential role for miR-146a in autoimmune response. Notably, miR-146a was found to be up-regulated in skin lesions of psoriasis, PBMC and synovial tissue of RA, whereas it was down-regulated in PBMC of systemic lupus erythematosus [20-22,25]. Although the increased expression of miR-146a in T cells does not show any affect on cytokine production in this study, available data on overexpression of miR-146a in several types of cells of RA suggests that miR-146a is possibly involved in modulating functions of T cells and other cells in RA pathogenesis. Although our current data confirm the increased miR-146a levels in synovial and peripheral blood CD4^+^T cells of RA patients, we were not able to differentiate the expression pattern of miR-146a levels in various CD4^+^T cell subpopulations due to the limited numbers of synovial and peripheral blood CD4^+^T cells. Further studies are warranted to clarify whether miR-146a expression is increased in activated T cells or preferentially upregulated in memory T cells of RA patients.

The current understanding of the critical roles of miRNAs in regulating cellular functions mainly depends on the identification of their target genes. TRAF6, a known target of miR-146a in macrophages and PBMCs, plays a key role in mediating signals from TNF receptor and IL-1 receptor in innate immunity. The suppressed TRAF6 expression by miR-146a indicated that miR-146a may be a negative regulator in the TNF-α signal pathway [22,34]. TRAF6 was also identified as a T cell-intrinsic negative regulator in mice with T cell-specific TRAF6 deletion, in which signs of hyperactive humoral immunity, including increased serum levels of immunoglobulin and DNA autoantibodies [37-39], were observed with similar clinical features of RA.

It has now become clear that certain miRNAs operate through targeting single genes while others act broadly through regulating the expression of multiple targets [40]. Up to now, the identification of new targets usually depends on bioinformatic analysis and experimental screening. Previously, gene expression analysis has facilitated the identification of targets upon overexpressed plant miRNAs [41]. In contrast to plants, vertebrate miRNAs are believed to exert their functions mainly through translational repression rather than mRNA cleavage [42]. However, it has been recently found that miRNAs can induce the degradation of mRNAs bearing fully complementary target sites by similar mechanisms of small-interfering RNA [43]. Transcriptome analysis can generate huge datasets with expression levels for all currently known genes.

Studies on the changes of mRNA expression induced by miRNAs have been considered as useful methods for uncovering their new targets and new downstream signal molecules [44,45], but only the degraded targets can be identified by transcriptome analysis. As many genes such as IRAK1, IRAK2, TRAF6, FADD, IRF-5, STAT-1 and PTC1 have been found to be targets of miR-146a in human disease [20,31,34,46], the targets and functions of miR-146a may be different in specific cells. In our experimental system, transcriptome analysis of miR-146a with up-or down-regulation in Jurkat T cells has not identified any known targets for miR-146a.

It is plausible to reason that miR-146a may mainly act through transcriptional repression rather than degradation of targets by imperfect basepair with the 3'-untranslated region of the targets. FAF1 is known to bind to the intracellular portion of the apoptosis signal transducing receptor Fas/Apo-1 and caspase-8 and shows similar characteristics of Fas-associated death domain protein, which can enhance Fas-mediated apoptosis [47,48]. We show that overexpression of FAF1 induces significant apoptosis in Jurkat T cells, consistent with previously reported findings [49]. Interestingly, the ectopic expression of miR-146a has been found to protect Jurkat T cells from activation-induced cell death, whereas FADD is identified as one of its targets [31]. Here, we further demonstrate that overexpression of FAF1 in miR-146a-overexpressing Jurkat T cells abolished the suppressive effect of miR-146a on T cell apoptosis. It becomes clear that activation-induced cell death is mainly mediated via the Fas/FasL pathway, which plays an important role in the immunity and induction of peripheral tolerance to self-antigens. A study by Ryu and colleagues has identified FAF1 as a member of Fas death-inducing signaling complexes such as FADD [50]. However, our transcriptome analysis of miR-146a failed to validate FADD as its target. Currently, it is unclear whether this is due to the detection limitation of the technique used or the possibility that miR-146a regulates FAF1 in an FADD-independent fashion. Interestingly, the expression of two targets of miR-146, TRAF6 and IL-1 receptor-associated kinase 1, remains unchanged although miR-146 is upregulated in PBMC from RA patients [22]. Thus, it remains to be elucidated whether miR-146 exerts its effect on various targets in a cell-type-dependent manner. Nevertheless, it is noteworthy that FAF1 does not seem to be a direct target of miR-146a according to our bioinformatics analysis [51]. Further studies are needed to identify genes involved in mediating the effect of miR-146a on FAF1 expression. Dysregulated T cell apoptosis is closely associated with autoimmunity diseases, especially RA. Thus, our findings that miR-146a upregulation in CD4^+ ^T cells is correlated with increased TNF-α levels may suggest that miR-146a acts as a critical factor in eliciting and maintaining the inflammation via suppressing T cell apoptosis during RA pathogenesis.

## Conclusions

In summary, this study revealed that miR-146a expression was upregulated while miR-363 and miR-498 were downregulated in CD4^+ ^T cells of RA patients. The level of miR-146a expression was positively correlated with levels of TNF-α, and *in vitro *studies showed TNF-α upregulated miR-146a expression in T cells. Moreover, we provided evidence that the increased expression of miR-146a suppressed T cell apoptosis by regulating FAF1. Our data indicate that increased miR-146a expression in CD4^+ ^T cells is possibly involved in maintaining inflammation during RA pathogenesis.

Although several independent studies have shown altered expression of miRNAs in PBMC or synovial tissue of RA patients, our findings that more cell-type-specific miRNAs were revealed by miRNA expression profile analysis also represent a distinct advantage. Up to now, the elucidation of miRNA function represents a veritable challenge for researchers. It seems that miR-146a regulate cell function through targeting multiple targets as several targets of miR-146a were identified. Thus the functions of miR-146a may be complex and diverse in specific cells or different stages of inflammation. Further studies on the role of miR-146a in modulating T cell function will not only increase our understanding of immune homeostasis, but also facilitate the identification of new therapeutic targets for the treatment of patients with RA.

## Abbreviations

bp: base pairs; DMARDs: disease-modifying antirheumatic drugs; FAF1: Fas-associated factor 1; FBS: fetal bovine serum; IFN: interferon; IL: interleukin; PBMC: peripheral blood mononuclear cell; miRNA: microRNA; RA: rheumatoid arthritis; SF: synovial fluid; TNF: tumor necrosis factor.

## Competing interests

The authors declare that they have no competing interests.

## Authors' contributions

JYL, YW and YZW designed and conducted all experiments and drafted the manuscript. QYG, LYZ, JYZ, JBZ and JJZ acquired and interpreted the miRNA data. FYF participated in assessing patients and in performing ultrasound guided joint aspirations. XLF and HLL acquired and analyzed the FACS data. LWL participated in the design of the study and interpretation of data, and was involved in drafting the manuscript. All authors have read and approved the final manuscript.

## Supplementary Material

Additional file 1 **Levels of Th1/Th2 cytokines in serum and SF of normal controls and RA patients**. Levels of Th1/Th2 cytokines in serum and synovial fluid (SF) of normal controls and rheumatoid arthritis (RA) patients. Levels of IL-2, IFN-α, IL-10, IL-4, IL-6 and TNF-α were up-regulated in SF of RA patients. Ctl -PB (n = 5): serum of healthy controls; RA-PB (n = 15): serum of RA patients; RA-SF (n = 36): synovial fluid of RA patient; **P < 0.01, *P < 0.05. Click here for file

Additional file 2 **Correlations between miRNAs and levels of cytokines in peripheral blood and SF of RA patients**. Expression of miR-498, miR-363 and miR-150 did not show any correlation with levels of cytokine (TNF-α, IL-2, IFN-α, IL-4, IL-6 and IL-10) production. Correlations between miRNAs (miR-498, miR-363, miR-150) and cytokines (TNF-α, IL-2, IFN-α, IL-4, IL-6 and IL-10) were analyzed by statistical evaluation and no significant results were found. Click here for file

Additional file 3 **Expression of various cytokines in freshly prepared CD4^+ ^T cells transfected with FUGW-miR-146a**. Flow cytometric analysis did not detect any significant alteration in the expression levels of IFN-γ, TNF-α, IL-2 and IL-17A in normal CD4^+ ^T cells transfected with miR-146a. Expression levels of IFN-γ, TNF-α, IL-2 and IL-17A were assayed by intracellular cytokine staining, but no significant change was detected between FF3-and miR-146a-CD4^+ ^T cells. Click here for file
