# Supplementary figures and images for: Altered microRNA expression profile with miR-146a upregulation in CD4+ T cells from patients with rheumatoid arthritis
Source: Arthritis Res Ther. 2010 May 11;12(3):R81. doi: 10.1186/ar3006 (PMC2911863; doi:10.1186/ar3006)

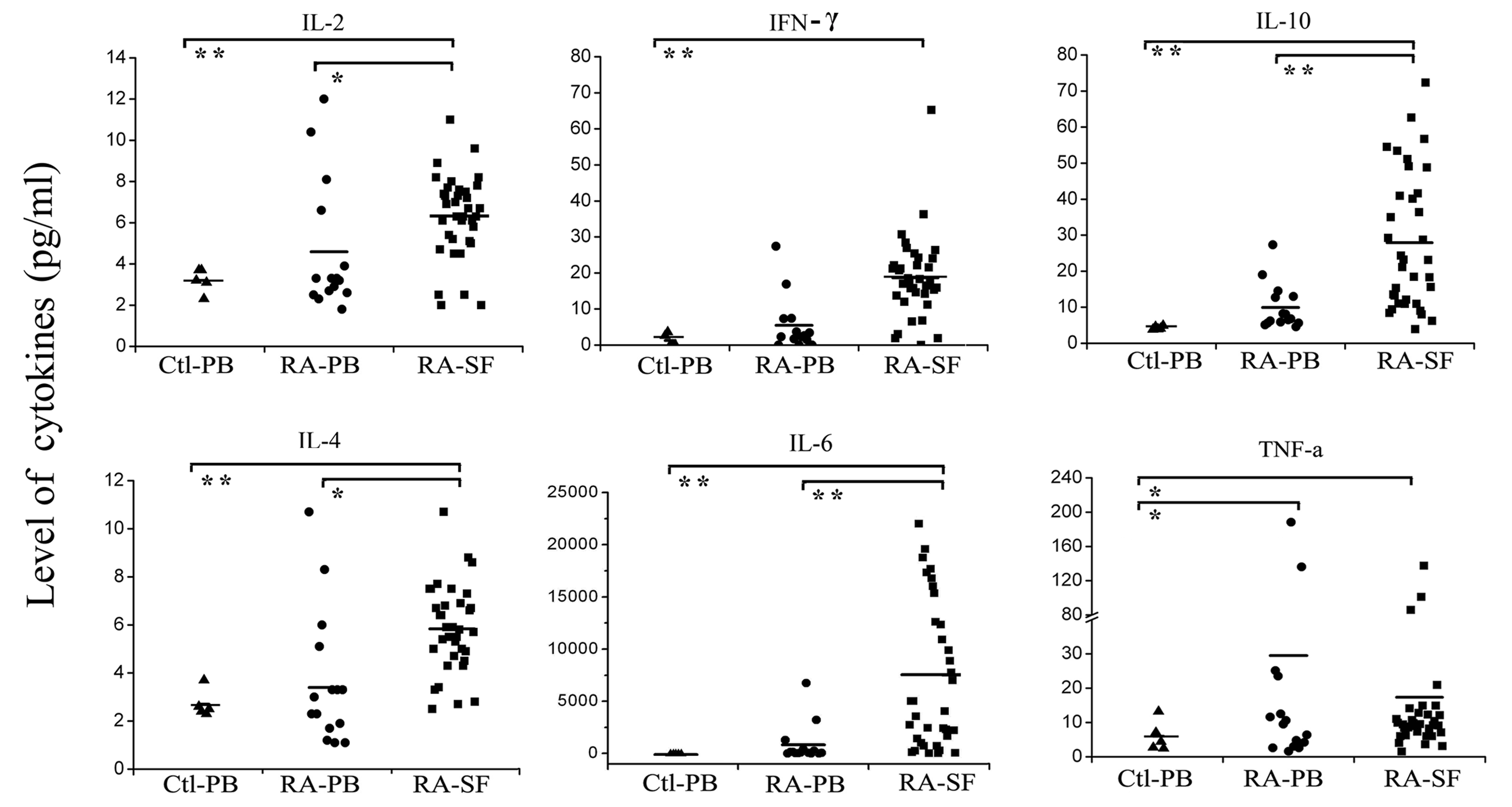

Supplement: Additional file 1 — Levels of Th1/Th2 cytokines in serum and SF of normal controls and RA patients. Levels of Th1/Th2 cytokines in serum and synovial fluid (SF) of normal controls and rheumatoid arthritis (RA) patients. Levels of IL-2, IFN-α, IL-10, IL-4, IL-6 and TNF-α were up-regulated in SF of RA patients. Ctl -PB (n = 5): serum of healthy controls; RA-PB (n = 15): serum of RA patients; RA-SF (n = 36): synovial fluid of RA patient; **P < 0.01, *P < 0.05. [file ar3006-S1.jpeg]

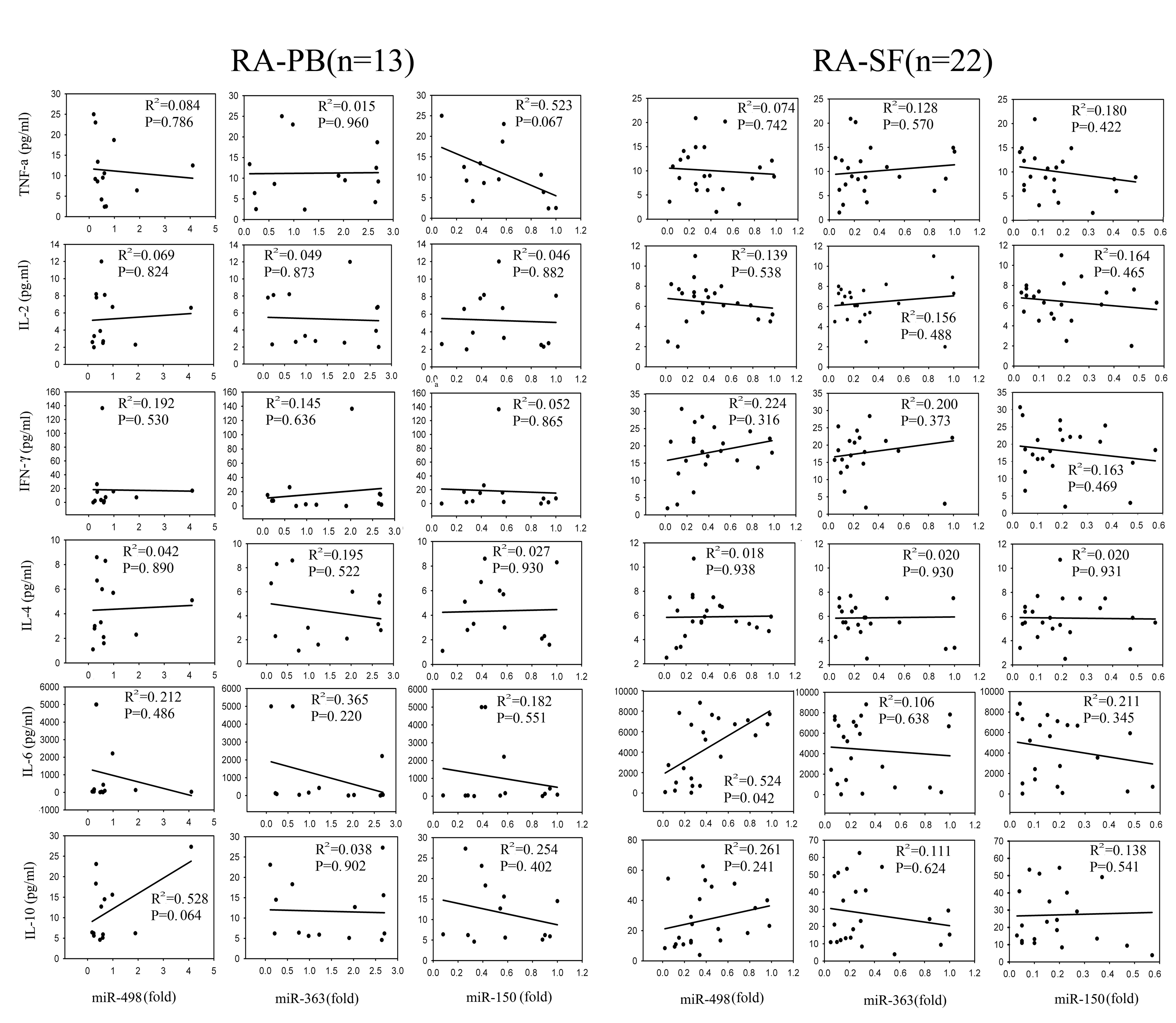

Supplement: Additional file 2 — Correlations between miRNAs and levels of cytokines in peripheral blood and SF of RA patients. Expression of miR-498, miR-363 and miR-150 did not show any correlation with levels of cytokine (TNF-α, IL-2, IFN-α, IL-4, IL-6 and IL-10) production. Correlations between miRNAs (miR-498, miR-363, miR-150) and cytokines (TNF-α, IL-2, IFN-α, IL-4, IL-6 and IL-10) were analyzed by statistical evaluation and no significant results were found. [file ar3006-S2.jpeg]

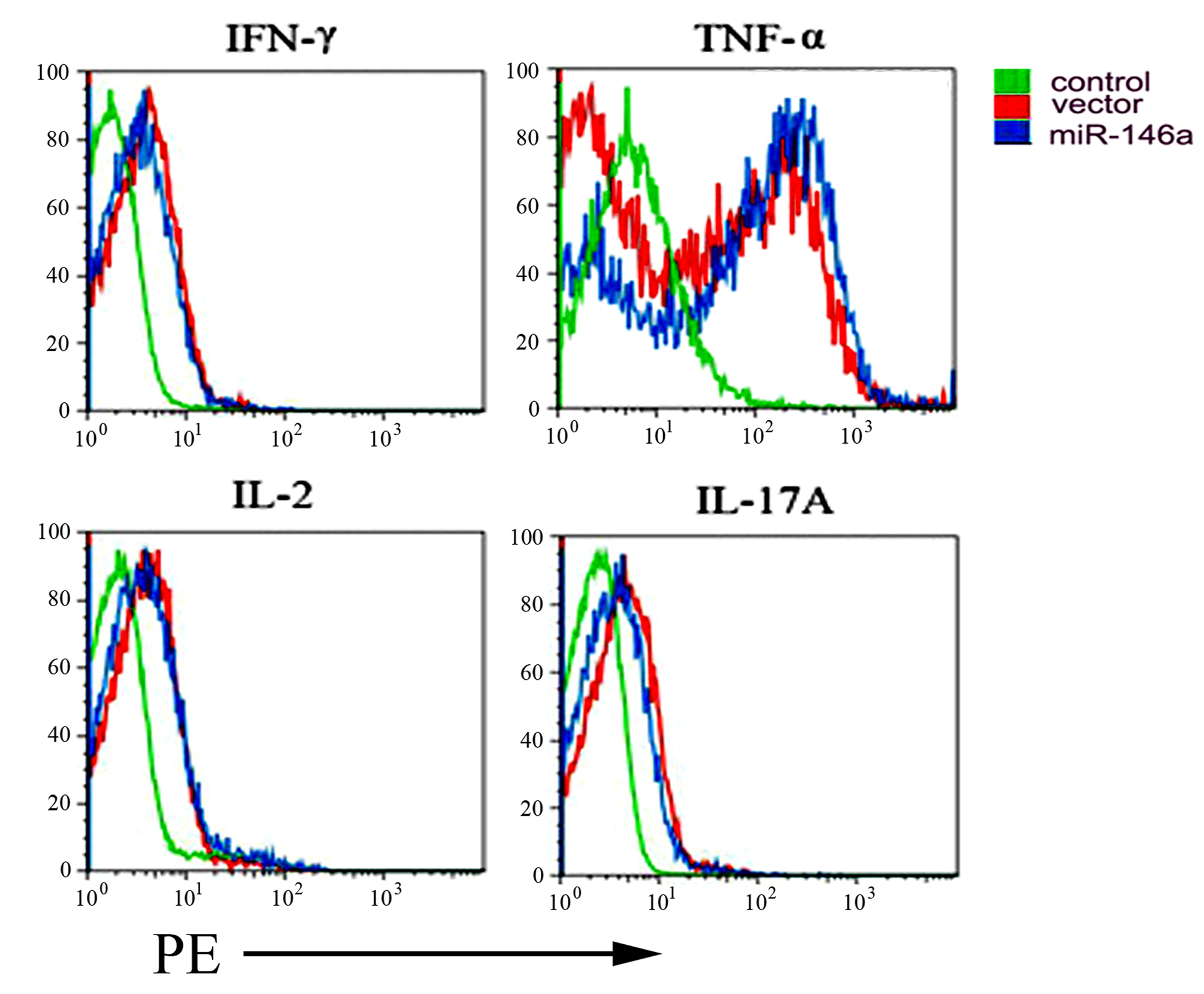

Supplement: Additional file 3 — Expression of various cytokines in freshly prepared CD4+ T cells transfected with FUGW-miR-146a. Flow cytometric analysis did not detect any significant alteration in the expression levels of IFN-γ, TNF-α, IL-2 and IL-17A in normal CD4+ T cells transfected with miR-146a. Expression levels of IFN-γ, TNF-α, IL-2 and IL-17A were assayed by intracellular cytokine staining, but no significant change was detected between FF3-and miR-146a-CD4+ T cells. [file ar3006-S3.jpeg]
